# Supplementary material for: Inhibiting Rho kinase promotes goal-directed decision making and blocks habitual responding for cocaine
Source: Nat Commun. 2017 Nov 30;8:1861. doi: 10.1038/s41467-017-01915-4 (PMC5707361; doi:10.1038/s41467-017-01915-4)
Supplement: Supplementary file 1 — Supplementary Information [file 41467_2017_1915_MOESM1_ESM.pdf]

Swanson Supplementary Materials

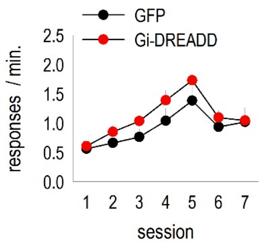

Supplementary Figure 1: Corresponds to primary text Fig.1. G_i-DREADDs, in the absence of CNO, do not impact instrumental response acquisition. Mice expressing G_i-coupled DREADDs (n=7) or GFP (n=12) do not differ when trained to nose poke for food reinforcers [no interaction F<1, main effect of session $F(6,102)=14.235, p<0.001$]. Infusion sites are represented in the main text, Fig.1. Bars and symbols = means + SEMs.

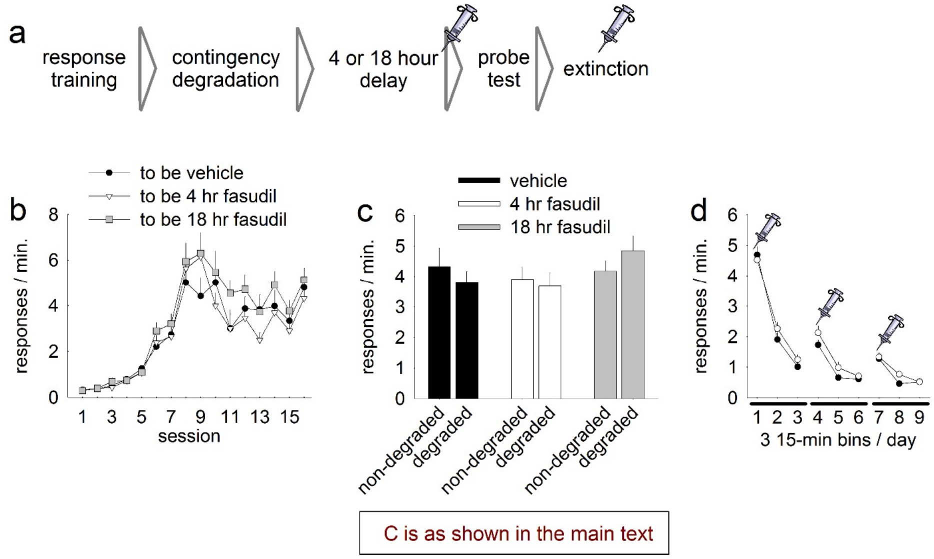

Supplementary Figure 2: Corresponds to primary text Fig.2. Delayed fasudil injection has no behavioral effects, and ROCK inhibition also does not influence response extinction. (a) Experimental timeline: To determine whether fasudil must be paired with an opportunity to learn new outcome-related information in order to enhance action-outcome conditioning, mice underwent identical response training as in main text Fig.2, but here, injections were delayed either 4 or 18 hours following instrumental contingency degradation. We also tested the effects of fasudil on extinction conditioning. (b) Groups (to be vehicle *vs.* to be fasudil) were designated by matching response rates. Vehicle groups did not differ and were combined [no interaction F<1, main effect of session $F(15,315)=51.930, p<0.001$, no effect of group $F(2,21)=1.124, p=0.344$] (n=8/group). (c) In a probe test following delayed injections, no groups responded preferentially, instead relying on habitual response strategies [no interaction F<1, no effect of response $F(1,42)=1.538, p=0.222$, no effect of group F<1]. (d) Fasudil also did not alter response extinction [no interaction F<1, main effect of session $F(8,176)=109.239, p<0.001$, no effect of group F<1]. Bars and symbols = means + SEMs. *p<0.05.

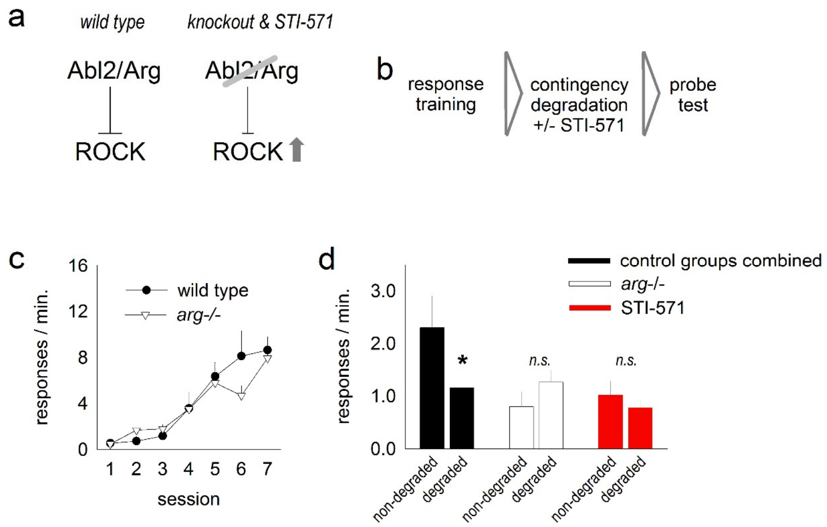

Supplementary Figure 3: Corresponds to primary text Fig.3. Ablating or inhibiting Arg kinase, an endogenous ROCK inhibitor, induces habit-based behavior. (a) In the brain, ROCK is endogenously suppressed by Abl2/Arg, such that ablating or inhibiting Arg disinhibits ROCK. (b) Experimental timeline: Mice with reduced levels of Arg, their wild type littermates, or intact C57BL/6 mice (for local infusion experiments) were trained to nose poke for food reinforcers, followed by instrumental contingency degradation and a probe test. (c) Arg knockout did not affect instrumental response acquisition [no interaction F<1, main effect of session F(6,54)=14.951,p<0.001, no effect of group F<1]. [In the case of local infusions, groups were determined by matching response rates during training and thus did not differ (all Fs<1; final day response rates, to be vehicle = 1.12±0.14, to be STI-571 = 1.21±0.11).] (d) Following instrumental contingency degradation, *arg*-/- mice and mice infused with STI-571 showed no response preference, indicating habit-based responding [interaction F(2,22)=5.663,p=0.01]. Response rates associated with the intact contingency were also lower in the *arg*-/- and STI-571 group [post-hoc p≤0.03 vs. control], an effect consistent with damage to the prelimbic cortex[1] and evidence that the prelimbic cortex is involved in motivated responding for food[2] (and reviewed[3]). Infusion terminals were within the prelimbic cortex, as in main text Fig.3. Bars and symbols = means + SEMs, *p<0.05.

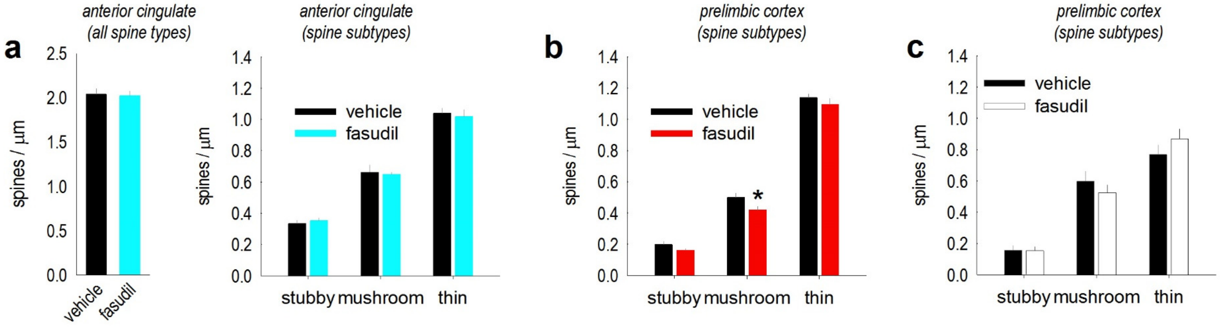

Mushroom-shaped spines in B are as shown in the main text

Supplementary Figure 4: Corresponds to primary text Fig.4. Fasudil selectively modifies dendritic spines. (a) Fasudil did not affect dendritic spine densities in the anterior cingulate cortex [t(15)=-0.205,p=0.840]. At right, dendritic spine subtypes are shown (also no differences). (b) In the prelimbic cortex, however, mushroom-shaped dendritic spine densities were lower in the fasudil group [t(15)=-2.357,p=0.032]. Other spine types were not affected. (c) When fasudil treatment was delayed 24 hours following modifications in instrumental contingencies, no significant effects were detected. Bars = means + SEMs, *p<0.05.

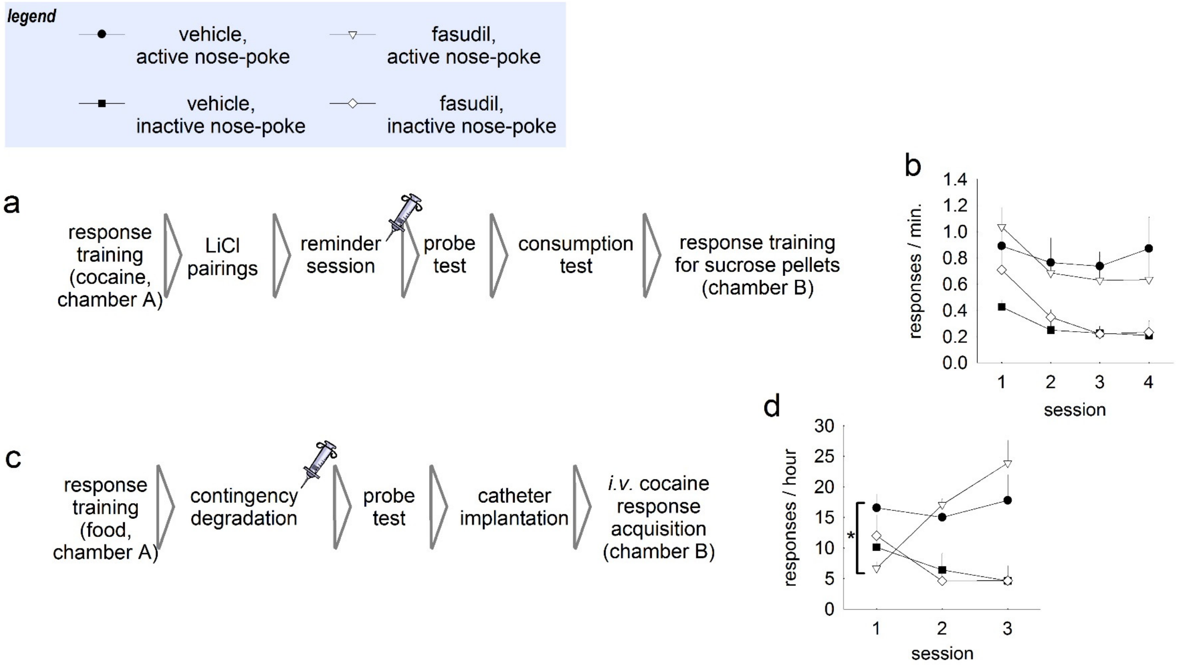

Supplementary Figure 5: Corresponds to primary text Fig.5. Fasudil selectively enhances sensitivity to action-outcome contingencies (as opposed to generally affecting response rates). (a) Experimental timeline: Mice were trained to respond for an orally-ingested cocaine-sucrose solution, followed by LiCl-induced CTA (see main text Fig.5). A brief “reminder session” was paired with vehicle or fasudil, followed by a probe test the following day and finally, a post-probe consumption test. These mice were then placed in distinct conditioning chambers and trained to respond for novel sucrose pellets. The two distinct training contexts are represented in Fig.5a of the main text. (b) Sucrose-reinforced response rates did not differ between groups, evidence that fasudil selectively enhanced sensitivity to the reduced value of the cocaine reinforcer and did not *generally* reduce response rates [no interaction $F(1,6)=2.402, p=0.172$, main effect of session $F(1,6)=17.069, p=0.006$, no effect of group $F<1$]. (c) Experimental timeline: Another cohort of mice originally generated to replicate our finding that fasudil enhanced sensitivity to action-outcome contingency degradation (see main text Fig.2b-d) was implanted with indwelling jugular catheters. (d) Here, a history of fasudil decreased cocaine-reinforced responding on day 1, likely because operant responding had recently been unpaired from reinforcer delivery, but *unlike* when fasudil was paired with the devaluation of cocaine, this group difference was quickly lost [interaction $F(2,12)=3.911, p=0.049$]. Bars and symbols = means + SEMs, *$p<0.05$.

Supplementary References

1. Corbit LH, Balleine BW (2003) The role of prelimbic cortex in instrumental conditioning. Behav Brain Res 146:145-157.
2. Gourley SL, Lee AS, Howell JL, Pittenger C, Taylor JR (2010) Dissociable regulation of instrumental action within mouse prefrontal cortex. Eur J Neurosci 32:1726-1734.
3. Pitts EG, Taylor JR, Gourley SL (2016) Prefrontal cortical BDNF: A regulatory key in cocaine- and food-reinforced behaviors. Neurobiol Dis 91:326-335.

Supplementary Table 1: Morphological measurements of dendritic spines

Corresponding to Figure 2	spine length (µm)	spine head diameter (µm)	
Saline	1.44 ± 0.021	0.38 ± 0.010	
Fasudil	1.37 ± 0.020	0.38 ± 0.009	
K-S test result	p=0.045	p=0.454	
Corresponding to Figure 4 *(prelimbic cortex, euthanized 1 hr after drug)*			
Saline	1.24 ± 0.013	0.32 ± 0.005	
Fasudil	1.28 ± 0.016	0.31 ± 0.005	
K-S test result	p=0.012	p=0.538	
Delayed saline	1.13 ± 0.014	0.41 ± 0.007	
Delayed fasudil	1.13 ± 0.013	0.38 ± 0.006	
K-S test result	p=0.907	p=0.023	
Corresponding to Figure 4 *(anterior cingulate cortex, euthanized 1 hr after drug)*			spine volume (µm^3)
Saline	1.03 ± 0.010	0.36 ± 0.004	0.15 ± 0.003
Fasudil	1.03 ± 0.010	0.35 ± 0.004	0.13 ± 0.003
K-S test result	p=0.525	p=0.378	p=0.004 (†)

Measurements of dendritic spine morphological parameters. Values refer to group means ± SEMs. Kolmogorov-Smirnov (K-S) tests were used to determine group differences. Each dendritic spine was considered an independent sample. $p<0.01$ but >0.001 was considered a trend, while $p<0.001$ was considered significant. † notes a trend for smaller spine volumes in the anterior cingulate cortex following fasudil.
